# The dramatic enhancement of ferromagnetism and band gap in Fe-doped In_2_O_3_ nanodot arrays

**DOI:** 10.1038/s41598-018-20751-0

**Published:** 2018-02-05

**Authors:** Feng-Xian Jiang, Dan Chen, Guo-Wei Zhou, Ya-Nan Wang, Xiao-Hong Xu

**Affiliations:** 1 School of Chemistry and Materials Science of Shanxi Normal University & Key Laboratory of Magnetic Molecules and Magnetic Information Materials of Ministry of Education, Linfen, 041004 China; 2 Research Institute of Materials Science of Shanxi Normal University & Collaborative Innovation Center for Shanxi Advanced Permanent Magnetic Materials and Techonology, Linfen, 041004 China

## Abstract

Ordered Fe-doped In_2_O_3_ nanodot arrays with diameters between 35 nm and 80 nm are fabricated using pulsed laser deposition with the aid of ultrathin porous anodized aluminumoxide templates. The 5 at.% Fe doped In_2_O_3_ nanodot arrays are shown to consist of the cubic bixbyite structure of In_2_O_3_. The nanodot arrays are demonstrated to be doped by Fe ions with mixed valences of +2 and +3, ruling out the presence of cluster and secondary phase related to Fe. The nanodot arrays exhibit the ferromagnetism at room temperature, where the magnetic moment increases as the dot size is reduced, rising to a maximum of about 230 emu/cm^3^ (equivalent to an average moment on the Fe ions of 15.30 µ_B_/Fe). This indicates an effect due to the surface of the nanodot arrays. The optical band width is also increased to 4.55 eV for the smallest dot array, thus indicating that the surface states are responsible for the magnetism and also enhance the band gap due to Burstein-Moss effect. Our results will be benefit for understanding the physical properties of oxide semiconductor nanostructures in the application of nano-spintronics devices.

## Introduction

Dilute magnetic semiconductors (DMSs), combining both semiconductor and ferromagnetic properties, have gained great attention due to their potential applications in spintronic devices^1^. Since Dietl *et al*. theoretically predicted the room temperature (RT) ferromagnetism in Mn doped ZnO and GaN semiconductors via Zener’s *p-d* exchange model^2^, many efforts have been carried out on a series of transition metal (TM) elements doped wide-band-gap oxide semiconductors, such as ZnO^3–5^, TiO_2_^6,7^, In_2_O_3_^8–13^ and SnO_2_^14,15^. Among them, Fe-doped In_2_O_3_ magnetic semiconductor is very attractive mainly due to its excellent electrical conductivity, high optical transparency, and high solubility of Fe ions in In_2_O_3_ host lattice^9,11^. In the past decade, lots of studies have mainly focused on the thin film, bulk, and polycrystalline powders of Fe-doped In_2_O_3_^8–10^. As advanced devices call for smaller nanostructured systems, the fabrication of lower-dimensional DMS structures is drawing increasing interest due to their advantages of small size, unique magnetic and optical properties^16,17^.

Actually, the fabrication and magnetic properties of Mn or Cr doped Ge, GaAs and InAs DMS nanostructured materials, such as quantum dots, have been reported^18–20^. In these studies, the self-assembly Stranski-Kranstanov (SK) growth method is adopted. However, this method results in randomly distribution, easily aggregate, and applies only to growing the material which has a large lattice mismatch with the substrate, such as MnGe and GaMnAs quantum dots on Si. Another method used to fabricate the well-patterned semiconductor nanostructures is the lithography technique or electron beam lithography, but they are impractical for large array sizes and cost relatively high^21,22^. Recently, ultrathin porous anodized aluminum (PAA) was used as contact templates to fabricate the large-areas, highly ordered nanostructures because of their low cost, more flexibility for pore size and separation control, easy fabrication, and so forth^23–26^. This bottom-up template can be used as the mask which excellently adheres to the substrate for direct deposition, and the highly ordered nanodot arrays can be obtained after removal the PAA templates.

In this paper, we presented the growth of DMS nanodot arrays by pulsed laser deposition (PLD) using the PAA templates, and provided the insights into the origin of the RT ferromagnetism in Fe-doped In_2_O_3_ nanodot arrays by a comprehensive analysis of structure and magnetic properties of the arrays. The band gap dependence on the size of Fe-doped In_2_O_3_ nanodot arrays was also investigated by the experiment.

## Results and Discussion

**Figure 1:**
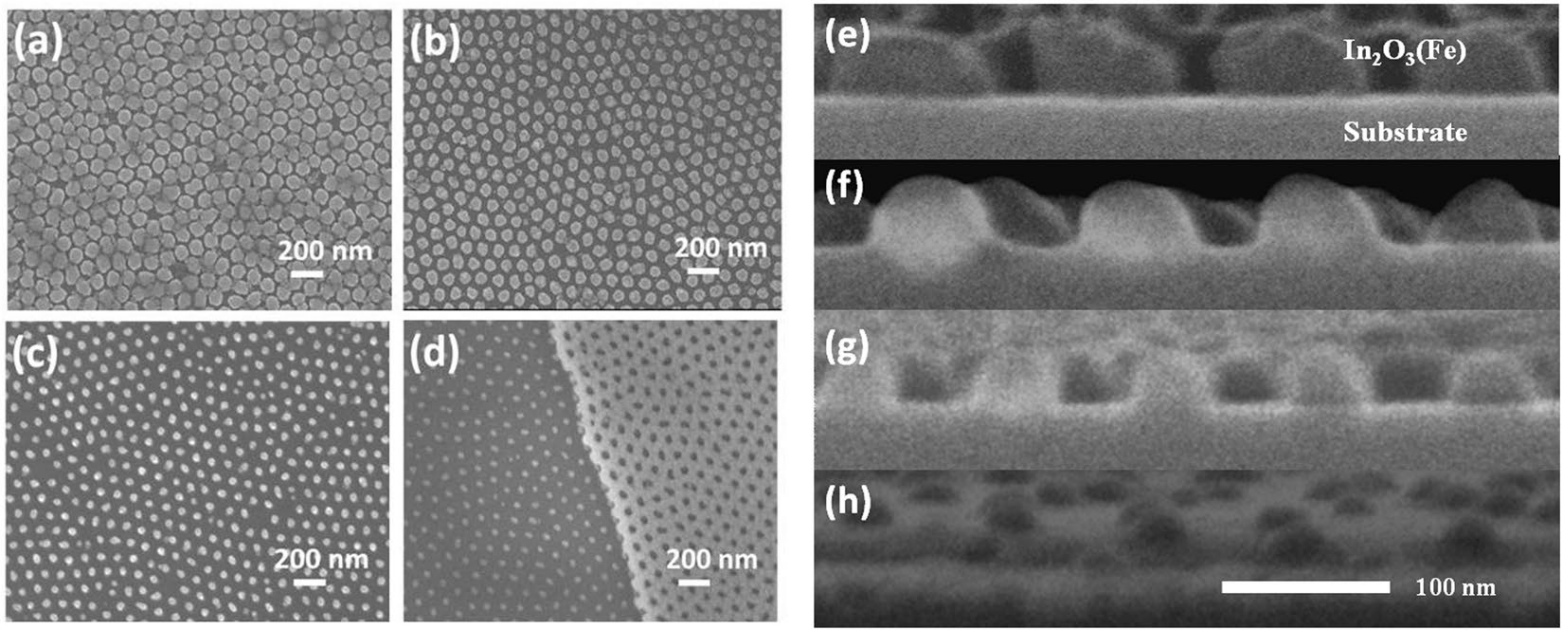
SEM images for the (In_0.95_Fe_0.05_)_2_O_3_ nanodot arrays. (**a**–**d**) The morphologies of nanodot arrays with average diameters of 80, 65, 50 and 35 nm and inter-dot distances of 105 nm. (**e**–**h**) The cross-sectional SEM images of corresponding nanodot arrays.

Figure 1(a–c) show the morphology of (In_0.95_Fe_0.05_)_2_O_3_ nanodot arrays with average diameters of 80, 65 and 50 nm, respectively. The dot height and inter-dot distance of these arrays are 40 nm and 105 nm, respectively. The highly ordered nanodot arrays are the same hexagonally lattice pattern as the PAA templates. The diameter distribution of the (In_0.95_Fe_0.05_)_2_O_3_ arrays is homogenous, and the diameters of these dot arrays are approximately equal to those of the corresponding templates. Moreover, the smaller diameter of nanodot arrays than that of the PAA templates can also be obtained by reducing the height of nanodot arrays. Figure 1(d) shows the morphology of (In_0.95_Fe_0.05_)_2_O_3_ nanodot arrays with the height of 10 nm and diameter of 35 nm, where the PAA template with the pore diameter of 50 nm is left behind intentionally. The AFM profiles (Supplementary Figure S2) also clearly show that the diameter of nanodot array is reduced with the decrease of its height.

Figure 1(e–h) show the cross-sectional SEM images of nanodot arrays with different average diameters, which clearly presents the different shape and size of the nanodot arrays. From the images, we can find that the nanodot arrays with diameters of 80, 65, 50 and 35 nm are cylinder-like shape, Mongolia package-like shape, hemi-ellipsoid-like shape, and dome-like shape, respectively. The difference of the nanodot arrays in shape are thought to arise from the closure effect during the deposition process due to the different aspect ratio (pore diameter over height) of the templates^27^.

**Figure 2:**
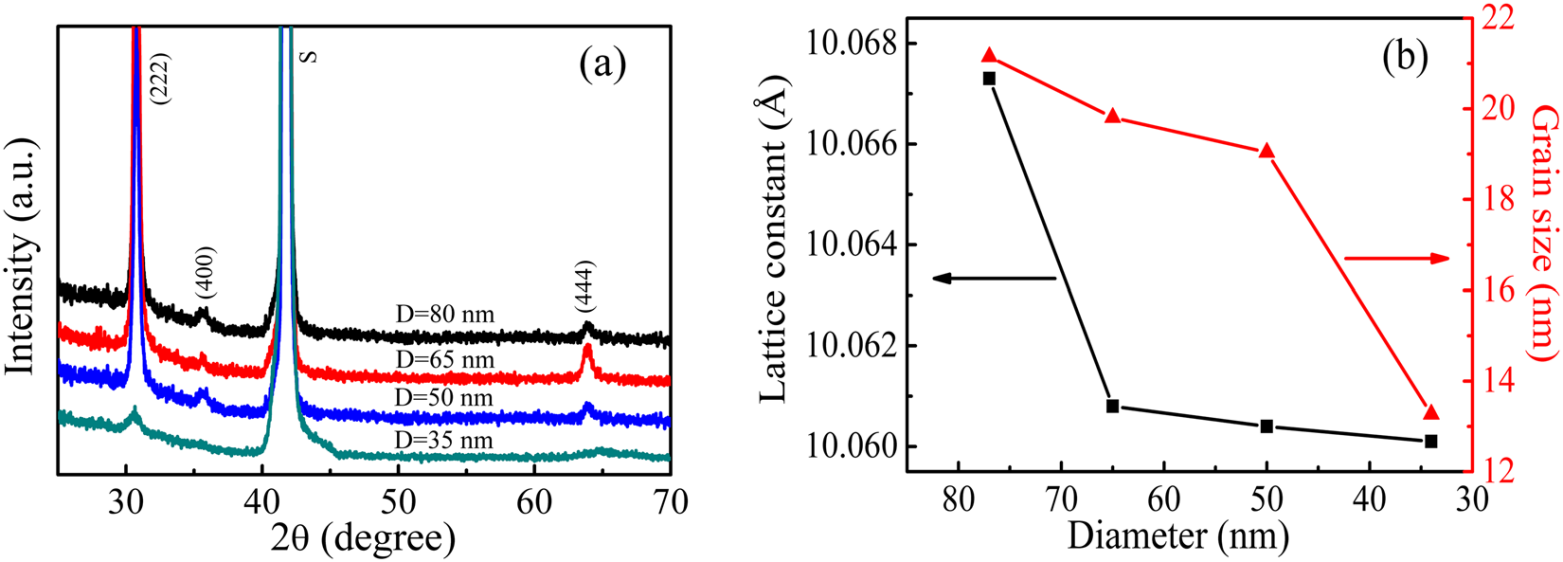
(**a**) The XRD patterns of (In_0.95_Fe_0.05_)_2_O_3_ nanodot arrays with different diameters, D. (**b**) The diameter dependence of lattice constant and grain size. The peaks marked with ‘S’ correspond to the peaks of Al_2_O_3_ substrates.

The diffraction patterns of the (In_0.95_Fe_0.05_)_2_O_3_ nanodot arrays with different diameters are shown in Fig. 2(a). All samples are indexed as cubic bixbyite structure of In_2_O_3_ and exhibit a preferred (222) orientation. No extra diffraction peaks of secondary phases, such as metallic Fe and Fe oxides, could be detected. A slight shift in the position of the (222) peaks to higher angles is observed, indicating a reduction in the lattice constant (see Fig. 2(b)), which might be due to the increased oxygen vacancies in the surface of the smaller nanodot arrays derived from their larger surface-to-volume^28^. It is also found that the (222) full width at half maximum (FWHM) peak of the nanodot arrays is large, and the FWHM becomes broader with the decrease of the diameter. This reveals the weak crystallinity and the small grain size of nanodot arrays. The grain size of (In_0.95_Fe_0.05_)_2_O_3_ nanodot arrays is also calculated by the Scherrer formula of d = 0.89λ/(β · cosθ) (*λ* is the wavelength of the x-ray, *θ* is the bragg angle, and *β* is the FWHM of 2*θ* in radians). As shown in Fig. 2(b), the grain size is decreased as the diameter reduces.

**Figure 3:**
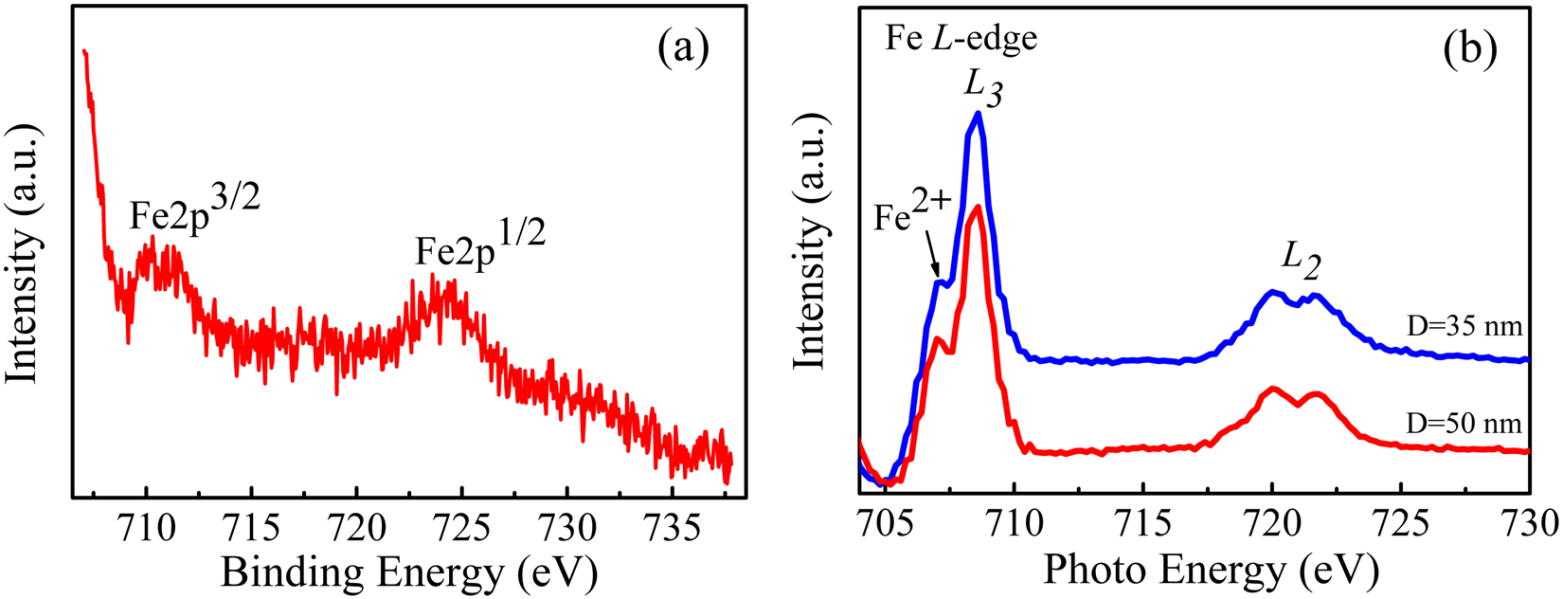
(**a**) The Fe 2p high-resolution XPS spectra for (In_0.95_Fe_0.05_)_2_O_3_ nanodot array with diameter of 50 nm. (**b**) The Fe L-edge XAS spectra for (In_0.95_Fe_0.05_)_2_O_3_ nanodot arrays with diameters of 50 nm and 35 nm.

The XPS measurement was performed to investigate the valence state of Fe element in the (In_0.95_Fe_0.05_)_2_O_3_ nanodot arrays. As shown in Fig. 3(a), the binding energy of Fe 2p^3/2^ and Fe 2p^1/2^ are located at 710.3 and 723.9 eV, respectively, which excludes the formation of metallic Fe clusters, the binding energy of Fe 2p^3/2^ and Fe 2p^1/2^ in which are respectively 707.0 and 720.0 eV^29^. Furthermore, comparing with the binding energies of Fe^2+^ 2p^3/2^ (709.9 eV) and Fe^3+^ 2p^3/2^ (711.0 eV)^29^, we can conclude that the Fe element exists in the ion form with the mixed valence of +2 and +3 in the (In_0.95_Fe_0.05_)_2_O_3_ nanodot array, which is likely facilitated by the oxygen vacancies due to the very low deposition oxygen pressure (5 × 10^−3^ mTorr). To further study the local structure of Fe in the In_2_O_3_ lattice, we performed the XAS measurements at the Fe *L*-edge. Figure 3(b) presents the normalized Fe *L*_*2*,3_ XAS spectra of the samples. It can be found that the *L*_*3*_ edge is composed of two peaks (707 eV and 709 eV) and 720 eV and 722 eV are related to the *L*_2_ edge. The split in the Fe *L*_*2*,3_ edges is usually attributed to the Fe ions with mixed valence of Fe^2+^ and Fe^3+^, further ruling out the appearance of metallic Fe^30,31^, which is consistent with the above XPS analysis. Moreover, as presented in the Fig. 3(b), there is a slight shift of Fe *L*_*3*_ edge shoulder peaks to lower energies with the decreased diameter of nanodot arrays, indicating an increase of the Fe^2+^/Fe^3+^ ratio. This increase of the Fe^2+^ ions in smaller nanodot arrays may be ascribed to the more oxygen vacancies due to the increase in surface-to-volume ratio as the diameter decreases^28^.

**Figure 4:**
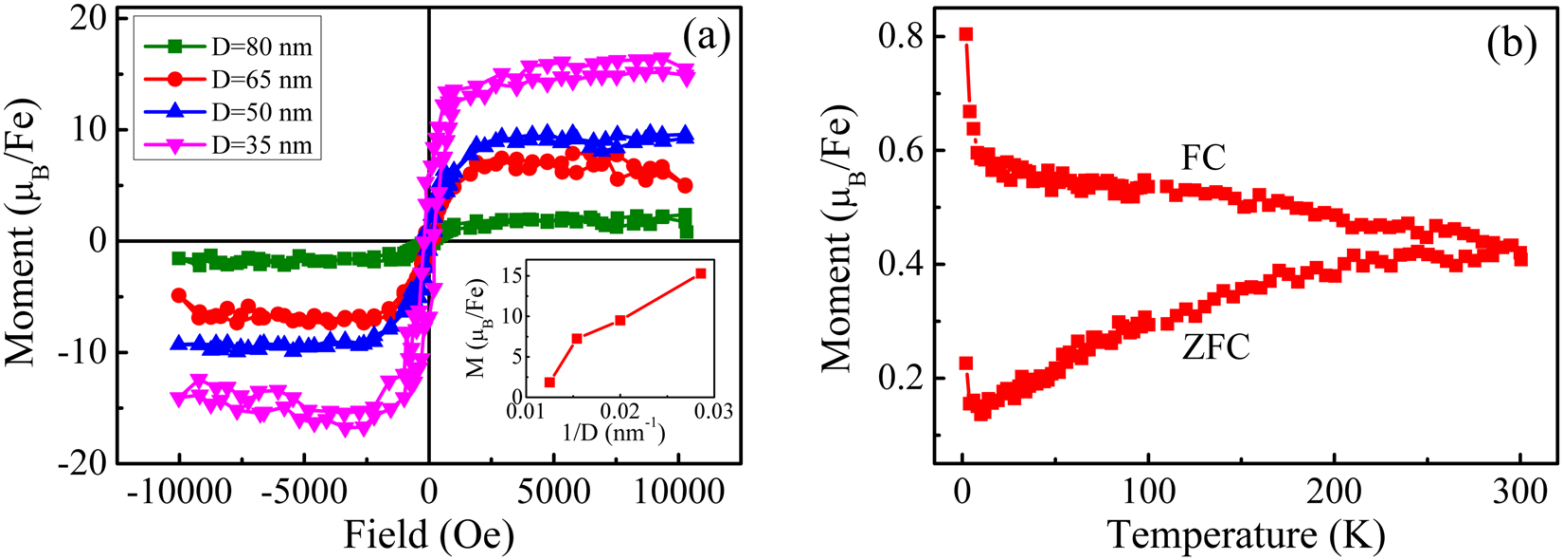
(**a**) Hysteresis loops measured at 300 K for (In_0.95_Fe_0.05_)_2_O_3_ nanodot arrays with different D. The inset shows the variation of M_s_ values with 1/D. (**b**) The FC/ZFC curves for the nanodot array with D of 50 nm measured at a field of 100 Oe.

All above structural and compositional results approve that the Fe element incorporates into the In_2_O_3_ host matrices by substituting the position of In atoms. In the following section, we will discuss the magnetic properties of our samples. Figure 4(a) shows the curves of magnetization versus magnetic field (M-H) when the external magnetic field is applied parallel to the sample surface (in-plane) after subtracting the diamagnetic background from the substrate (Supplementary Figure S3). The well-defined hysteresis loops can be clearly observed for all (In_0.95_Fe_0.05_)_2_O_3_ nanodot arrays, indicating a strong ferromagnetism above RT. As shown in Fig. 4(a), the saturation magnetic moment (M_s_) of nanodot arrays sharply increases from 1.85 to 15.30 µ_B_/Fe as the nanodot size decreases (Noted that the calculated dot volume is based on its surface and cross-section morphology in order to reduce calculate error, which is shown in Supplementary), which is much larger than that of the 5 at.% Fe doped In_2_O_3_ films^8,11^. Such huge M_s_ in zero dimensional nanodot arrays may be attributed to the abundant defects associated with the larger surfaces since the defects are reported to play an important role in the magnetic behavior of DMSs^31^. It is known that the vacancies and interstitials in oxide semiconductors can occur during the growth^32^. For zero-dimensional nanostructures, such defects are more prevalent as compared to films because of the increase in the surface area^33^. As the nanodot size decreases, the surface defects are increased due to the increase of the surface-to-volume ratio, and therefore, the enhanced magnetic moments is observed. Plot of M_s_ (µ_B_/Fe) vs 1/D shown in the inset of Fig. 4(a) indicates the importance of surface states to the magnetism in the (In_0.95_Fe_0.05_)_2_O_3_ nanodot arrays.

The zero-field cooled (ZFC) and field-cooled (FC) curves for the 50 nm-diameter nanodot array is shown in Fig. 4(b). The obvious divergence of the ZFC and FC curves indicates that the samples are ferromagnetic in the whole temperature range. The blocking temperature do not appears in the ZFC curves, suggesting that the tiny ferromagnetic nano-clusters, such as metallic Fe and Fe oxides nanoparticle, can be ruled out^34^. This agrees with our results of XRD, XPS and XAS.

**Figure 5:**
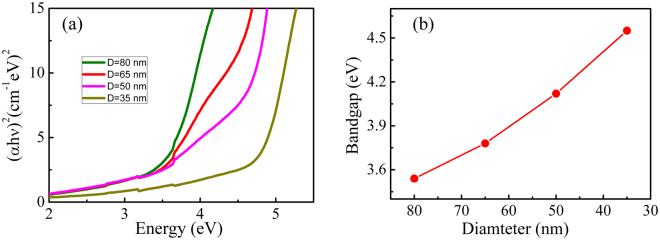
(**a**) Dependence of α^2^ on photon energy for (In_0.95_Fe_0.05_)_2_O_3_ nanodot arrays with different D. (**b**) The band gap of nanodot arrays as a function of the diameter.

Figure 5(a) shows the absorption spectra of the (In_0.95_Fe_0.05_)_2_O_3_ nanodot arrays with different diameters. With the decrease of the nanodot arrays in diameter, the absorption edge of nanodot arrays shows an obvious blue-shift, indicating an increase of the band gap. Figure 5(b) shows the optical band gap determined by the Tauc model^35^:

1 $$\,{({\rm{\alpha }}{\rm{h}}{\rm{\nu }})}^{2}={\rm{A}}\,\ast \,({\rm{h}}{\rm{\nu }}-{{\rm{E}}}_{{\rm{g}}})$$

where *α* is the absorption coefficient; *hν* is the photo energy; *E*_*g*_ is the optical band gap; and *A* is a constant. The *E*_*g*_ of the samples is obtained by extrapolating the linear region to zero absorption. It can be seen that the band gap increases from 3.54 to 4.55 eV as the diameter decreases from the 80 to 35 nm. The increased band gap in (In_0.95_Fe_0.05_)_2_O_3_ nanodot arrays can be possibly attributed to the Burstein-Moss effect (BM effect). The BM shift of the *E*_*g*_ can be obtained by the following equation:^36^

2 $$\Delta {{\rm{E}}}_{{\rm{BM}}}=\frac{{h}^{2}}{8{m}_{e}^{\ast }}{(\frac{3}{{\rm{\pi }}})}^{2/3}{n}_{e}^{2/3}$$

where ${m}_{e}^{\ast }$, *n*_*e*_, and *h* are the effective electron mass in the conduction band, carrier concentration and Plank’s constant, respectively. As the diameter of nanodot arrays gets smaller, as mentioned above, there will be more oxygen vacancies which act as donors and contribute to carriers. Obviously, an increase in the carrier density will ultimately induce a blue-shift of the band gap. The decrease of the lattice constant and grain size shown Fig. 2(b) will also contribute a wider band gap^37,38^.

## Conclusions

In summary, we have observed the RT ferromagnetism in the highly ordered Fe-doped In_2_O_3_ nanodot arrays with different sizes. The Fe-doped In_2_O_3_ nanodot arrays exhibit the obvious size-dependent magnetic and optic behaviors. Moreover, the surface defects play a crucial role in the unique huge magnetic moment of the Fe-doped In_2_O_3_ nanodot arrays. The increased band gap in (In_0.95_Fe_0.05_)_2_O_3_ nanodot arrays is possibly attributed to the Burstein-Moss effect. These results are very promising for future spintronic devices.

## Experimental Details

In our experiment, the ultrathin PAA templates with various pore diameters were fabricated by a two-step anodization at a constant voltage of 40 V in 3.6 wt% H_2_C_2_O_4_ at 0 °C. After the two-step anodization, a thin polymethylmetacrylate (PMMA) layer was spin-coated onto the top surface of the PAA templates, and then the aluminum was etched away by immersing the template in a mixture of CuCl_2_ and HCl. The thin nonporous barrier layer was removed by floating the PAA templates in 5 wt% H_3_PO_4_ at 30 °C for 40 min. The template was transferred to the Al_2_O_3_ substrate, and the PMMA layer was dissolved by C_3_H_6_O at 60 °C. In this work, the average pore diameter of the PAA templates can be tuned from 50 to  80 nm by immersing the PAA templates in H_3_PO_4_ for different durations, and the thickness and inter-pore distance of the mask was about 200 and 105 nm, respectively. The Fe-doped In_2_O_3_ nanodot arrays was subsequently deposited on the PAA/Al_2_O_3_ (0001) substrate from the ceramic target of nominal composition (In_0.95_Fe_0.05_)_2_O_3_ by the PLD using a KrF excimer laser (λ = 248 nm) with a pulse repetition rate of 10 Hz and an energy density of 250 mJ/pulse. The target-to-substrate was 7 cm and the ceramic (In_0.95_Fe_0.05_)_2_O_3_ target was prepared from high-purity In_2_O_3_ (99.995%, Alfa Aesar) and α-Fe_2_O_3_ (99.995%, Alfa Aesar) powders by a conventional solid-state reaction technique. During deposition, the substrate temperature was maintained at 600 °C, the chamber oxygen pressure was 5 × 10^−3^ mTorr, and the height of nanodots were controlled by the deposition time. After deposition, the samples were cooled down to room temperature slowly at the same oxygen pressure. The PAA template was then dissolved using a 5 wt% NaOH solution at 35 °C and highly ordered (In_0.95_Fe_0.05_)_2_O_3_ nanodot arrays were obtained.

The morphology of the (In_0.95_Fe_0.05_)_2_O_3_ nanodot arrays and PAA templates were investigated by scanning electron microscope (SEM) and atomic force microscopy (AFM). The crystal structures of the nanodot arrays and the films were characterized by x-ray diffraction (XRD) using Cu K_α_ radiation (λ = 0.15406 nm). The composition of the samples was determined by x-ray photoelectron spectroscopy (XPS) and x-ray absorption spectra (XAS). Optical transmission spectra of the samples were measured by an UV-vis spectrophotometer. The magnetic properties measurements were performed by a superconducting quantum interference device (SQUID) magnetometer.

## Electronic supplementary material

Supplementary Information
