# Supplementary material for: The dramatic enhancement of ferromagnetism and band gap in Fe-doped In2O3 nanodot arrays
Source: Sci Rep. 2018 Feb 5;8:2417. doi: 10.1038/s41598-018-20751-0 (PMC5799379; doi:10.1038/s41598-018-20751-0)
Supplement: Supplementary file 1 — Supplementary Information [file 41598_2018_20751_MOESM1_ESM.docx]

**Supporting Information**

**The dramatic enhancement of ferromagnetism and band gap in Fe-doped In_2_O_3_ nanodot arrays**

**Feng-Xian Jiang, Dan Chen, Guo-Wei Zhou, Ya-Nan Wang & Xiao-Hong Xu**

We prepared ultrathin PAA templates using the two-step anodization process. Before anodization, the high-purity aluminum foils were cleaned with acetone, ethanol, and distilled water in turn, and then annealed at 500 ℃ for 2 hours to remove the mechanical stresses. The Al foils were then eletropolished in a electrolyte consisting of HClO_4_ and C_2_H_5_OH with the volume ratio of 1:4. After that, the processed Al foils were first anodized at a DC voltage of 40 V in a 0.3 M H_2_C_2_O_4_ solution at 0℃ for 2 hours. After removing the oxidized Al layer, the second anodization process was performed at the same parameter as the first anodization. The unoxidized Al layer was removed in saturated CuCl_2_ solution, and the remaining alumina layer was immersed again in 5wt% H_3_PO_4_ to remove the barrier layer. Finally, we obtain the ultrathin PAA templates. The PAA templates with different pore diameters can be obtained by varying the dipping time in 5wt% H_3_PO_4_. Figure S1 shows the PAA templates with diameter of 50, 65 and 80 nm.


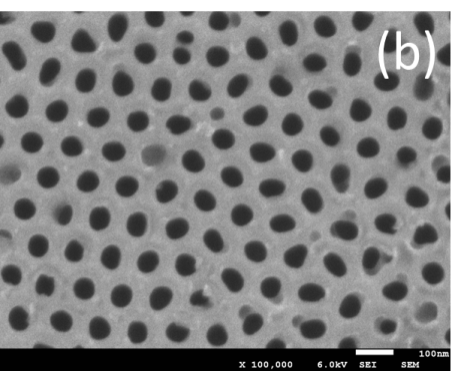

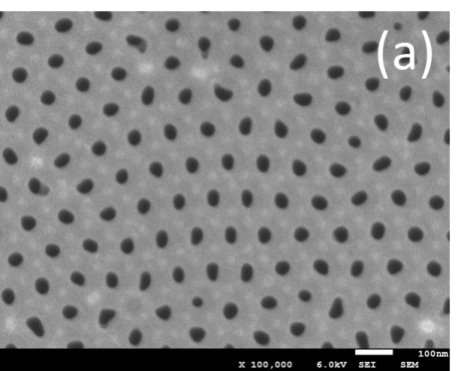

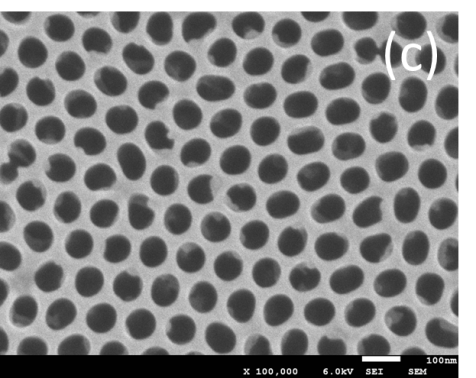


Figure S1. SEM images of PAA templates with diameter of (a)-(c) 50, 65 and 80 nm, respectively.

(a) (b)


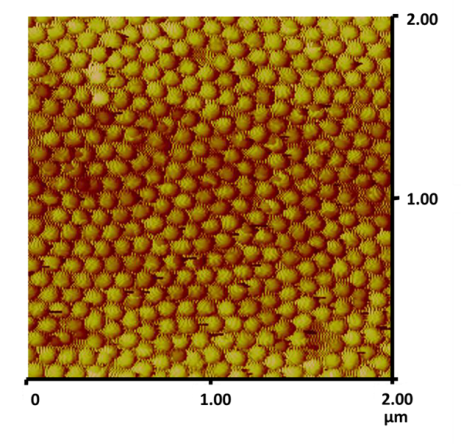

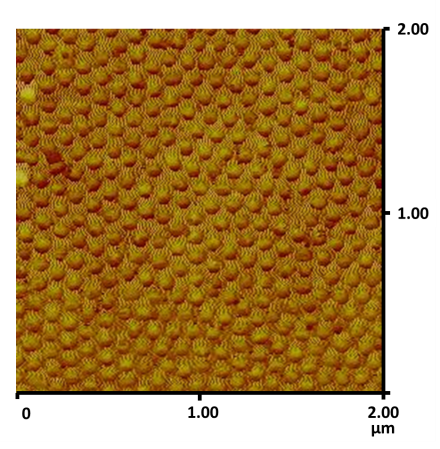

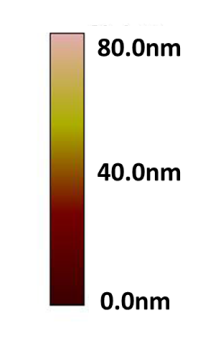


Figure S2. AFM images of Fe-doped In_2_O_3_ nanodot arrays with diameters and heights of (a) 50, 40 nm; (b) 40, 20 nm.


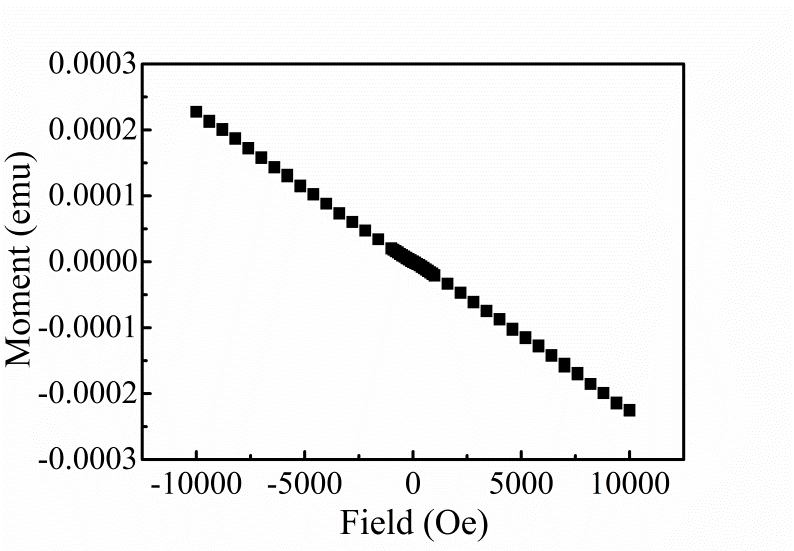


Figure S3. The M-H curve of Al_2_O_3_ (0001) substrate.

Figure S4 shows the hysteresis loops measured at 5 and 300 K for (In_0.95_Fe_0.05_)_2_O_3_ nanodot array with the diameter of 50 nm. The M_s_ decreases from 11.5 to 9.5 µ_B_/Fe as the temperature increases from 5 to 300 K. The coercive fields are respectively 150 Oe and 65 Oe at 5 and 300 K, which is a typical behavior of ferromagnetic material.


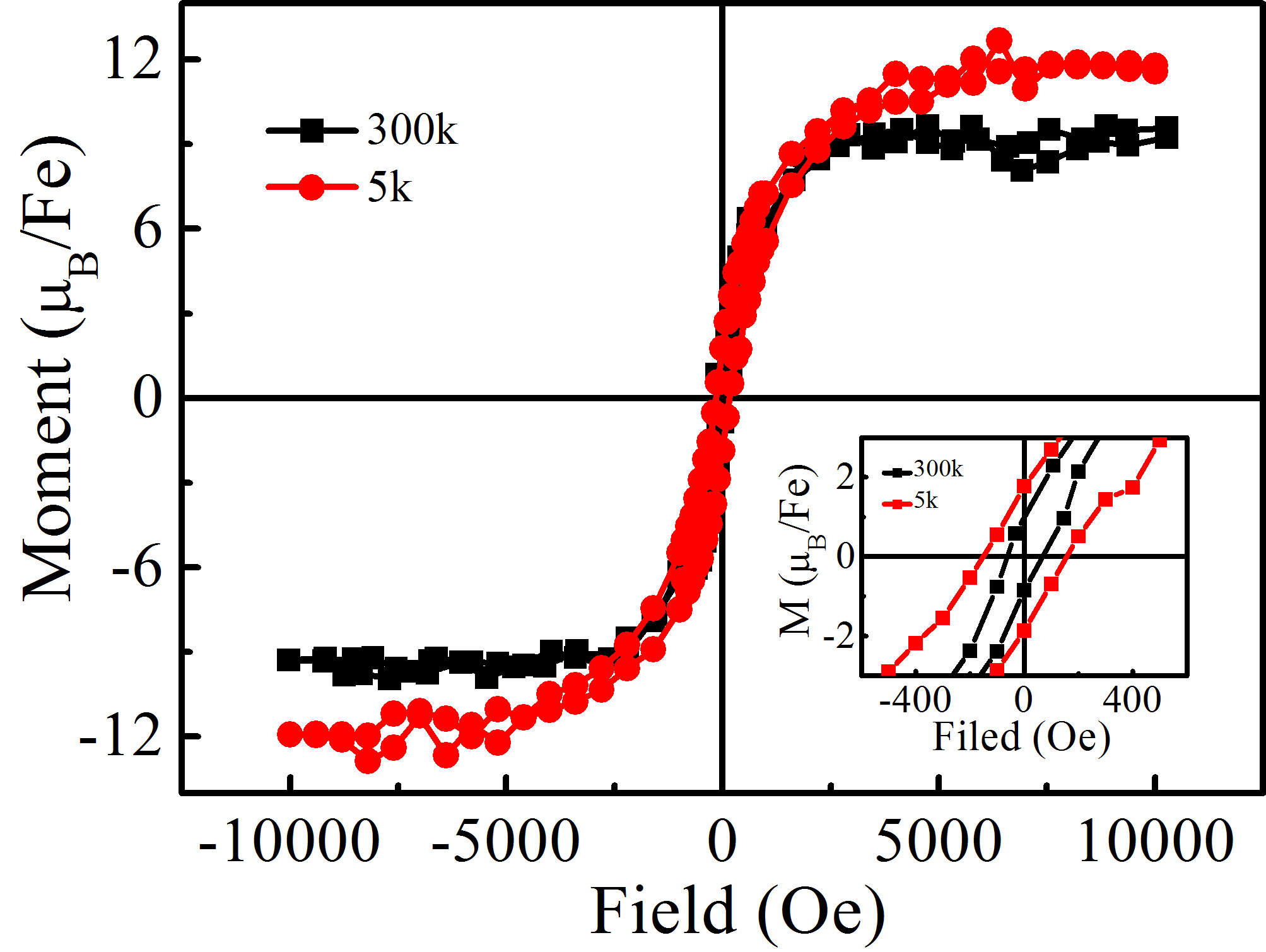


Figure S4 shows the hysteresis loops measured at 5 and 300 K for (In_0.95_Fe_0.05_)_2_O_3_ nanodot array with the diameter of 50 nm. The insets showed the hysteresis loops on the expanded scale.

In the experiment, the volume of the nanodot arrays was estimated using the formula:

$$V= \frac{2}{3} \pi\left( \frac{D}{2} \right)^{2}H S \rho$$

by assuming the shape of the nanodots is hemispherical. In this formula, D and H are the diameter and height of the nanodot arrays, respectively, and they can be obtained from the SEM images of the nanodot arrays (Figure 1); S is the area of the Al_2_O_3_ substrate (about 0.25 cm^2^) and ρ is density of nanodot arrays. The ρ was evaluated using the formula:

$$\rho= \frac{2 x {10}^{14}}{\sqrt{3}D_{\mathrm{int}}^{2}} \mathrm{cm}^{-2}$$

where D_int_ is the interpore distance. The density is calculated to be 1.05 x 10^10^ cm^-2^ for a AAO mask with the D_int_ of 105 nm.
